# Nutrition of the Critically Ill—A 21st-Century Perspective

**DOI:** 10.3390/nu5010162

**Published:** 2013-01-14

**Authors:** Stig Bengmark

**Affiliations:** Division of Surgery & Interventional Science, University College London, 4th floor, 74 Huntley Street, London, WC1E 6AU, UK; E-Mail: stig@bengmark.se; Tel.: +44-20-7511-6842

**Keywords:** health care, surgery, stress, trauma, transplantation, liver cirrhosis, liver steatosis, obesity, osteoarthritis, pancreatitis, critical care, nutrition, enteral nutrition, parenteral nutrition, microbiota, microbiome, microbial translocation, probiotic bacteria, lactobacillus, lactobacillus plantarum, lactobacillus paracasei, microbial translocation, inflammation, infection, Toll-like, neutrophils, pharmaceuticals, biological, eco-biologicals, nutraceuticals, curcumin, resveratrol, antibiotics, chemotherapeutics, barriers, leakage, gut, airways, oral cavity, skin, vagina, placenta, amnion, blood-brain barrier, growth, replication, apoptosis, mucosa, endothelium, plaques, cytokines, IL1, NF-kB, TNF, growth factors, insulinogenic, IGF-1, prebiotics, plant fibers, greens, fruits, vegetables, minerals, fat diet, refined carbohydrate diet, advanced glycation end products (AGEs), advanced lipoxidation end products (ALEs), endotoxin, LPS, proteotoxins, casein, gluten, zein, western lifestyle, paleolithic lifestyle, food habits of the chimpanzee

## Abstract

Health care-induced diseases constitute a fast-increasing problem. Just one type of these health care-associated infections (HCAI) constitutes the fourth leading cause of death in Western countries. About 25 million individuals worldwide are estimated each year to undergo major surgery, of which approximately 3 million will never return home from the hospital. Furthermore, the quality of life is reported to be significantly impaired for the rest of the lives of those who, during their hospital stay, suffered life-threatening infections/sepsis. Severe infections are strongly associated with a high degree of systemic inflammation in the body, and intimately associated with significantly reduced and malfunctioning GI microbiota, a condition called dysbiosis. Deranged composition and function of the gastrointestinal microbiota, occurring from the mouth to the anus, has been found to cause impaired ability to maintain intact mucosal membrane functions and prevent leakage of toxins—bacterial endotoxins, as well as whole bacteria or debris of bacteria, the DNA of which are commonly found in most cells of the body, often in adipocytes of obese individuals or in arteriosclerotic plaques. Foods rich in proteotoxins such as gluten, casein and zein, and proteins, have been observed to have endotoxin-like effects that can contribute to dysbiosis. About 75% of the food in the Western diet is of limited or no benefit to the microbiota in the lower gut. Most of it, comprised specifically of refined carbohydrates, is already absorbed in the upper part of the GI tract, and what eventually reaches the large intestine is of limited value, as it contains only small amounts of the minerals, vitamins and other nutrients necessary for maintenance of the microbiota. The consequence is that the microbiota of modern humans is greatly reduced, both in terms of numbers and diversity when compared to the diets of our paleolithic forebears and the individuals living a rural lifestyle today. It is the artificial treatment provided in modern medical care—unfortunately often the only alternative provided—which constitute the main contributors to a poor outcome. These treatments include artificial ventilation, artificial nutrition, hygienic measures, use of skin-penetrating devices, tubes and catheters, frequent use of pharmaceuticals; they are all known to severely impair the microbiomes in various locations of the body, which, to a large extent, are ultimately responsible for a poor outcome. Attempts to reconstitute a normal microbiome by supply of probiotics have often failed as they are almost always undertaken as a complement to—and not as an alternative to—existing treatment schemes, especially those based on antibiotics, but also other pharmaceuticals.

## 1. Morbidity and Death Continue to Rise in Modern Medicine

Despite some breathtaking advances in medico-pharmaceutical and advanced surgical treatments, in the chronically ill, as well as in various emergency situations, health care is still affected by unacceptably high morbidity and mortality rates. The main cause of a poor outcome is often infections/sepsis, today often referred to as health care-associated infections (HCAI). The incidences of HCAI have increased dramatically during recent decades, and seem to continue to do so. In fact, HCAI constitutes one of the fastest, if not *the* fastest, growing and unsolved problems in modern medicine. With the present rate of increase, it has the potential to at least double in incidence by the year 2050. Sepsis is estimated to each year affect at least 18 million individuals worldwide, and mortality rates are expected to reach 25% to 30% [1,2]; severe sepsis is calculated as killing more individuals annually than prostate cancer, breast cancer, and HIV/AIDS combined, and the numbers of cases affected by sepsis are creeping up from year to year [3]. 

The increase in HCAI has occurred much in parallel to, and is strongly associated with, the increased use of invasive technologies; it is currently reported as constituting the fourth leading cause of disease in industrialized countries [4]. More than 230 million major surgical procedures are estimated to be undertaken each year worldwide [5]. It has been calculated that approximately 25 million patients worldwide will each year undergo high-risk surgery, and no less than 3 million will not make it home [6]. A recent study followed 46,539 adult patients undergoing standard inpatient non-cardiac surgery at 498 hospitals across 28 European nations. Four percent of the included patients died before discharge, a significantly higher mortality rate than expected [6]. The lowest rates were observed in Estonia, Finland, Iceland, Norway, the Netherlands and Sweden, and the highest were registered in Belgium, Croatia, Ireland, Italy, Latvia, Poland, Romania and Slovakia. These findings are strongly correlated with the access to critical care in these countries. As a matter of fact, most of those who died (73%) had never been admitted to critical care at any stage in connection with the surgical procedure, and almost half (43%) of those treated in the ICU had been returned to standard wards before dying [6].

## 2. Health Care-Associated Infections (HCAI) Do Not Receive Enough Attention

Complications after surgical procedures remain a leading cause of death [7,8,9,10], and, unfortunately, they are continuously increasing. Furthermore, patients who develop complications but survive to leave the hospital will still continue to suffer reduced functional independence and also suffer reduced long-term survival [7,11,12,13]. About 10% of the patients who today undergo surgery are known to develop complications, and about 80% of all postoperative deaths are currently registered [8,9,10]. It is of the greatest importance that the characteristics of these patients, and the risk of various treatments, are analyzed in detail.

Recent observations in the US suggest that not only the number of incidences, but also the severity of sepsis, have significantly increased during the last decades [14,15,16]. Modern advanced surgery carries a high rate of septic morbidity and especially esophageal, pancreatic, and gastric procedures are particularly known to represent great risk for the development of sepsis. Thoracic, adrenal, and hepatic procedures have been identified as bearing the highest sepsis-induced mortality rate [14,15]. It is documented in the literature that elderly patients, men, and nonwhites are more likely to develop sepsis as a complication to surgical treatment [14,15]. Sepsis is by far the most common medical and surgical complication, and estimated only in the US annually to affect as many as 751,000 [17,18], and cause the death of approximately 215,000 patients (29% of all treated patients) [18], making sepsis the tenth most common cause of death in this country.

## 3. Artificial Nutrition—A Major Contributor to Sepsis

Obesity and various other chronic diseases are especially known to be associated with poor outcome. The highly artificial environment of modern ICUs, the extensive use of pharmaceuticals, artificial nutrition, assisted ventilation, in addition to the use of skin-penetration devices, *etc.* are all strongly associated with poor outcome. A recent multivariate analysis identified emergency surgery, mechanical ventilation, fluid resuscitation, and use of vasoactive drugs in the postoperative period as the strongest indicators of risk of sepsis [19]. Other studies suggest use of artificial feeding regimens, both enteral and parenteral, as major contributors to ICU-associated sepsis; catheter-related sepsis is reported to occur in about 25% of patients fed via intravenous feeding-tubes [20]. Other common perioperative practices, e.g., preoperative antibiotics [21], and mechanical bowel preparation [22,23] will often, instead of the expected prevention of infections, lead to significantly increased rates of treatment-associated infections. Other ICU measures such as mechanical ventilation [24], use of various pharmaceutical drugs, antibiotics [25,26], hemo-therapeutics, chemical solutions for clinical nutrition, and a number of pharmaceuticals, contribute significantly to the high degree of systemic inflammation and, indirectly, to sepsis. 

The trauma/operation-induced acute phase response and increased inflammatory reaction in the body is most often aggravated by the supply of various pharmaceuticals which seemingly paralyze important immune functions, and impair important neutrophil and macrophage functions. The inflammation is made worse by aggressive supply of nutrients, especially when used parenterally, but also when applied enterally. Herndon *et al.* [27] reported in 1989, in their now-seminal study in burn patients, a great disadvantage of feeding the patients via the parenteral (PN) route instead of enteral (EN); identical solutions were either supplemented as PN or EN, and a dramatic difference in mortality rate was observed; 63% after PN compared to 26% after EN (*p* < 0.05). It is increasingly recognized that hyperalimentation should no longer be routine practice, at least not during the first 10–14 days after surgery. Administration of larger amounts of fluid and electrolytes [28,29,30], fat [31,32,33], sugars [34,35,36], macromolecules/colloids, dextrans [37] and hydroethylstarch (HES) [38] demonstrated to increase immune dysfunction, reduce resistance to disease, and increase morbidity. 

## 4. Colloid-Associated Morbidity Often Neglected

We demonstrated already 35 years ago in an experimental study comparing the effects of 0.9% NaCl solution, dextran 70, hydroxyethyl starch, degraded gelatin and fat emulsion, that dextran 70, hydroxyethyl starch, and degraded gelatin caused significant hemodilution and decreased platelet count [39]. Our subsequent studies demonstrated that dextrans, degraded gelatin and hydroxyethyl starch all increased APT time, impaired ADP- and collagen-induced aggregation, and induced a significantly increased bleeding time and blood loss after experimental liver resection [40]. Another study of ours at that time demonstrated that dextrans significantly increased mortality in experimental pneumococcal infections (59%) and numbers of abscesses compared to animals treated with only saline (23%) (*p* < 0.05) [41]. When the susceptibility to induced pneumococcal peritonitis was studied after supply of dextrans or fat emulsions (Intralipid), both induced significantly higher mortality; dextrans 80% (*p* < 0.01), fat emulsions 47% (*p* > 0.05) compared to saline (20%) [42].

It is not easy to understand that both HES and dextrans continue to be used despite the fact that a Cochrane study already in the year 2000 found no advantages of them over crystalloids. A recent report under the auspices of the European Society of Intensive Care Medicine (ESICM) analyzed the experience of colloid treatment in mixed intensive care units (ICU), especially in cardiac surgery, head injuries, sepsis and organ donor patients, as reported in recent meta-analyses, systematic reviews and clinical studies, concluding: “*We recommend not to use colloids in patients with head injury and not to administer gelatins and HES in organ donors. We suggest not using hyperoncotic solutions for fluid resuscitation. We conclude and recommend that any new colloid should be introduced into clinical practice only after its patient-important safety parameters are established*” [38]. It should especially be avoided in liver surgery, particularly in liver resections with its dramatic reduction in numbers of immune cells and parenchymal liver cells. 798 patients with severe sepsis were in a study performed by the Scandinavian Critical Care Trials Group randomly assigned in the ICU to fluid resuscitation with either 6% HES 130/0.42 (Tetraspan^®^, Braun Medical Supplies) or Ringer’s acetate (Sterofundin ISO^®^, BraunMedical Supplies); the HES-supplied patients reported to suffer a significantly increased risk of death within three months and also to be significantly more likely to require renal-replacement therapy, as compared with those receiving only Ringer’s acetate [43].

## 5. Crystalloids Often Enough after Surgical Procedures

In fact, and as observed already about 15 years ago, large amounts of calories seem not to be needed during the first 10–14 days after common surgery or trauma, clearly shown in two well-designed randomized studies involving 300 and 195 resp., patients, who underwent major general surgery in Scandinavia [44], and in the US [45], respectively. In the first study, which lasted for up to 15 days, the supply of total parenteral nutrition (TPN) was compared to infusion of only 1000–1500 kcal/day of glucose. The nitrogen loss during the first week was reduced to about half in the glucose-supplied group compared to the TPN group, but no differences in morbidity or mortality were observed. The authors thus concluding that “*overfeeding seems to be a larger problem than underfeeding*” [44]. In the second study, performed at Memorial-Sloan-Kettering Cancer Center in New York, the patients were randomized to either early enteral supplementation with a so-called immuno-enhancing diet (Impact^®^, Novartis) or only IV crystalloid infusions. The daily intake of calories was low in both groups, 1000 kcal and 400 kcal, that is, 61% and 22% of defined goals (25 kcal/kg/day). No differences in the number of minor, major or infectious complications, number of wound infections, mortality or length of stay were observed between the groups [45]. 

Numerous experimental and clinical studies have demonstrated that enteral nutrition formulas are commonly deleterious to the immune functions and, as observed, will reduce microbiota and impair gut barrier functions. It was demonstrated in experimental animals already 20 years ago that various commercial clinical nutrition formulas will almost immediately upon enteral supply induce the loss of intestinal barrier function, promote bacterial translocation, and impair host immune defense [46], a phenomenon that is today often observed in humans. These experimental studies demonstrated a dramatic increase in incidence of bacterial translocation to the mesenteric lymph node when the animals were fed nutrition formulas such as Vivonex^®^ (53%), Criticare^®^ (67%), or Ensure^®^ (60%) (*p* < 0.05) [25,46,47]. Significant elevations in pro-inflammatory are reported in patients after pancreat-duodenectomy when fed a standard enteral nutrition solution (Nutrison^®^); IL-1beta day 7 (*p* < 0.001); day 14 (*p* = 0.022), TNF-alpha—day 3 (*p* = 0.006); day 7 (*p* < 0.001) [48]. It is of special interest that such changes were no longer observed when the standard nutrition was replaced by a formula [48], claimed to have immune-modulatory effects (Stresson^®^); instead, anti-inflammatory cytokines were observed to be significantly elevated: IL-1ra/s: day 7 (*p* < 0.001); IL-6: day 10 (*p* = 0.017); IL-8: day 1 (*p* = 0.011) days 3, 7, 10, and 14 (*p* < 0.001), and IL-10: days 3 and 10 (*p* < 0.001) [48]. 

## 6. Nutrition Made to Prevent Deterioration of Immune Functions

The tolerance to LPS of commercial diets was studied in experimental animals divided into the following three groups of diet: (1) control diet receiving standard soy-based diet rich in cysteine, crude fibers and ω-6 PUFA linoleic acid but devoid of eicosapentaenoic acid (EPA) and docosahexaenoic acid (DHA); (2) a 100% whey-peptide-based commercially available liquid diet (Peptaman AF, Nestlé) high in cysteine, as well as in EPA, DHA and prebiotic fructooligosaccharides (FOS); and (3) a casein-based liquid isonitrogenous diet (Promote^®^ Very High Protein Nutrition, Abbott), low in cysteine and devoid of EPA, DHA and FOS. The whey-peptide-based diet rich in EPA-DHA, cysteine and FOS protected the animals significantly better than the two other diets against specific and general effects of systemic inflammatory syndrome, but also against damage to tissues, particularly the liver [49].

The effect of early enteral nutrition with a new immune-modulatory diet (IMD) enriched with hydrolyzed whey peptide (HWP), a protein complex derived from milk, and suggested to have anti-inflammatory effects, was recently studied (MHN-02, MEIN^®^, Meiji Dairies Co., Tokyo, Japan) in 40 patients after living-donor liver transplantation (LDLT). The treated patients demonstrated, when compared to 36 patients who received a conventional elemental diet (Elental^®^, Ajinomoto Pharma Co., Tokyo, Japan) (control group), a considerably reduced incidence in bacteremia in the HWP group (15%) than in the control group (47%) (*p* = 0.002). Although it did not reach statistical significance, a reduced incidence of infection-induced mortality was also reported (*p* = 0.145) [50].

## 7. Dysbiosis-Associated Over-Reacting Neutrophils

Dysbiosis-associated systemic inflammation is almost regularly observed in severe trauma and after surgery, accompanied by severe leakage of endotoxin into the body, and leading to infection and sometimes severe sepsis. As a consequence, there is a significant decrease in lymphocytes, and a significant, often disproportionate, increase in both circulating and tissue neutrophils, paralleled by a persistent decline in T-4 helper lymphocytes and elevation of T-8 suppressor lymphocytes [51]. It is suggested that a T-4/T-8 lymphocyte cell ratio of <1 is a sign of severe immunosuppression and prediction of poor outcome in conditions such as multiple and severe trauma, multiple organ dysfunction syndrome, severe acute pancreatitis and in myocardial infarction and stroke, as well as during chemotherapeutic treatments, particularly in oncology patients [52]. 

A large early increase in circulating neutrophils is always accompanied by tissue infiltration of neutrophils, responsible for common post-trauma/postoperative dysfunctions such as paralytic ileus [53,54], bone marrow suppression, endothelial cell dysfunction, and responsible for tissue destruction and organ failure, particularly in the lungs [55,56,57,58], intestines [55], liver [59] and kidney [60]. Neutrophil infiltration to distant organs [61], particularly the lungs [57], is significantly aggravated by mechanical therapeutic efforts such as handling of the bowels during operation [53], and ventilation of the lungs [62]. Poor nutritional status, preexisting immune deficiency, obesity, diabetes and in high levels of blood sugar [63] contribute to immune deterioration and to increased expressions of molecules such as NF-κB, COX-2, LOX and iNOS [63,64].

It is important to remember that a disproportionate increase in circulating neutrophils can, to a large extent, be successfully inhibited by the supplementation of antioxidants [65,66,67], as well as specific probiotics [68,69]. Supplementation of probiotics is also shown to effectively prevent neutrophil infiltration of the lung and reduce subsequent tissue destruction, as demonstrated in studies with inflammation induced by ceacal ligation and puncture (CLP)—please see below. 

## 8. Efforts to Reduce Inflammation, Neutrophil Infiltration, and Tissue Destruction

**Figure 1:**
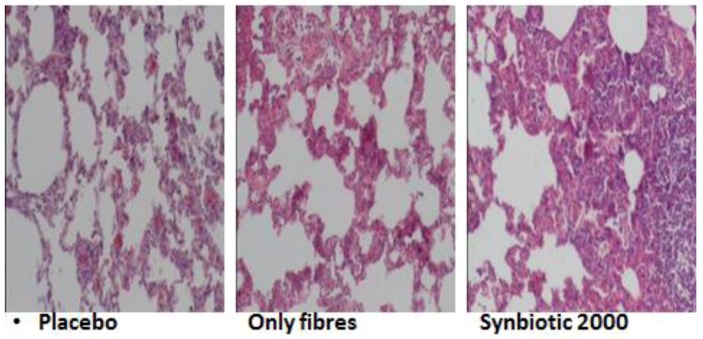
Hematoxylin-eosin of lung tissues from placebo, only fibers-treated and Synbiotic 2000-treated animals [71].

Experimental animals, subjected to induced infections through ceacal ligation and puncture (CLP), were treated with prophylactic supplementation using a synbiotic cocktail, Synbiotic 2000 Forte (see below). The treatment consisted in supplementation of a cocktail of the four LAB, which either was injected subcutaneously at the time of trauma [70], or supplied orally together with prebiotic fibers as a pretreatment for three days before induction of trauma [71]. Both treatments effectively prevented neutrophil accumulation in the lung tissues (Table 1) as well as pulmonary tissue destruction (Figure 1). Significant reductions in parameters associated with the degree of systemic inflammation, such as myeloperoxidase (MPO, Table 2), malondialdehyde (MDA, Table 3) and nitric oxide (NO, Table 4), indicated a significant suppression of trauma-induced inflammation, all differences between the treatment and placebo groups in the two studies being statistically significant (*p* < 0.05) [71]. 

**Table 1:** Neutrophil counts after treatment with Synbiotic 2000, only the LAB in Synbiotic 2000, only the fibers in Synbiotic 2000 and placebo [71]; *p* < 0.05.

Treatment group	Average number of neutrophils per viewed fields
**Synbiotic 2000**	**9.00 ± 0.44**
Only LAB	8.40 ± 0.42
Only the fibers	31.20 ± 0.98
Placebo	51.10 ± 0.70

**Table 2:** Myeloperoxidase (MPO) activity in the supernatant presented as U/g lung tissue, after treatment with Synbiotic 2000, only the LAB in Synbiotic 2000, only the fibers in Synbiotic 2000 and placebo, respectively [71]; *p* < 0.05.

Treatment group	U/g
**Synbiotic 2000**	**25.62 ± 2.19**
Only LAB	26.75 ± 2.61
Only the fibers	56.59 ± 1.73
Placebo	145.53 ± 7.53

**Table 3:** Lipid peroxidation in the lung tissue determined expressed as levels of malondialdehyde (MDA), measured in nmol/mg protein, after treatment with Synbiotic 2000, only the LAB in Synbiotic 2000, only the fibers in Synbiotic 2000 and placebo respectively [71]; *p* < 0.05.

Treatment group	nmol/mg
**Synbiotic 2000**	**0.22 ± 1.31**
Only LAB	0.28 ± 3.55
Only the fibers	0.48 ± 5.32
Placebo	0.67 ± 2.94

**Table 4:** Lung tissue nitrite (NO2) and nitrate (NO3), expressed as µmol/g wet tissue, after treatment with Synbiotic 2000, only the LAB in Synbiotic 2000, only the fibers in Synbiotic 2000 and placebo, respectively [71]; *p* < 0.05.

Treatment group	µmol/g
**Synbiotic 2000**	**17.16 ± 2.03**
Only LAB	8.91 ± 2.24
Only the fibers	47.71 ± 3.20
Placebo	66.22 ± 5.92

## 9. Life-Threatening Systemic Inflammation

A study of patients in intensive care suffering life-threatening extreme systemic inflammation, which is called “systemic inflammation response syndrome” (SIRS), and its relation to gut microbiota, was recently published. Gut microbiota in twenty-five patients with severe SIRS and a level in serum of C-reactive protein above 10 mg/dL were analyzed [72] and were found to bear markedly lower total anaerobic bacterial counts, particularly of the beneficial Bifidobacterium and Lactobacillus, paralleled by higher counts of total facultative anaerobes such as Staphylococcus and Pseudomonas compared to healthy volunteers. Gram-negative facultative anaerobes were the most commonly identified microbial organisms in mesenteric lymph nodes and at serosal scrapings at laparotomy. Gastrointestinal complications were strongly associated with a significantly reduced number of total obligate anaerobes and highly increased numbers of Staphylococcus and Enterococcus and significantly decreased numbers of total obligate anaerobes and total facultative anaerobes [72]. 

A more recent study in 63 similar patients suggests impaired gastrointestinal motility as a significant marker of poor outcome [73]. Patients with ≥300 mL per day reflux from nasal gastric feeding tube demonstrated significantly lower numbers of total obligate anaerobes including Bacteroidaceae and Bifidobacterium, higher numbers of Staphylococcus, lower concentrations of acetic acid and propionic acid, and higher concentrations of succinic acid and lactic acid (*p* ≤ 0.05), accompanied by dramatically higher incidences of bacteremia (86% *vs.* 18%) and mortality (64% *vs.* 20%) than patients without gastric distension (*p* ≤ 0.05) [73]. Furthermore, in 29 similar patients, treatment with a synbiotic composition, consisting of *Bifidobacterium breve *and *Lactobacillus casei*, in combination with galactooligosaccharides, was attempted. Higher levels of *Bifidobacteria* and *Lactobacillus*, but also total organic acids, particularly short-chain fatty acids, were reported and the incidence of infectious complications such as enteritis, pneumonia, and bacteremia, compared to historical controls, was reported to be significantly lower in the treated group [74]. 

## 10. Numerous Mechanisms to Control Intestinal Homeostasis

Dysbiosis and impaired barrier functions are associated with several negative consequences; translocation of lipopolysaccharides (LPS) and whole microbial cells, accumulation of endotoxin in the body (endotoxemia) and hyperactivation of the immune system. The microbiota controls intestinal homeostasis through numerous mechanisms in which substances such as lipopolysaccarides, flagellins, peptidoglycans, and formulated peptides are involved. It interacts with intestinal cell receptors such as Toll-like receptors and activates important intracellular signaling pathways with the ability to modulate processes such as cell survival, replication and apoptosis, as well as inflammatory response. Among the challenging molecules are NF-κB, caspases, mitogen-activated protein kinases. The host immune system controls microbial composition through release of molecules such as β-defensins, cryptidins, lectins, angiogenin 4, reactive oxygen species, IgA and so-called bacteriocins, which effectively limits the expansion of various pathogenic microorganisms [75,76]. 

The enteric flora is mostly represented by strict anaerobes (70%–90%), which predominate over facultative anaerobes and aerobes (10%–30%) [76]. Recent studies suggest that the gut microbiota might be classified as belonging to one of three principal variants, or “enterotypes” defined by a dominant presence of *Bacteroides*, *Prevotella*, or *Ruminococcus* species [77]. However, increasing evidence suggests that these enterotypes are more microbial gradients than, although discrete, defined microbial communities as most of the observed differences are largely explained on the basis of long-term dietary intake [78,79]. Diet has the most powerful influence on gut microbial communities in healthy human subjects [80,81,82]. A study of human subjects and 59 other mammals revealed clusters in which the effects of diet (carnivorous, omnivorous, or herbivorous) almost always outweigh host phylogeny [80]. *Bacteroides *species are prevalent with long-term protein and animal fat diets, whereas *Prevotella *species are associated with long-term carbohydrate diets [82]. 45,000 of the presently identified >800,000 rDNA sequences (microbial species) and about five of the about 50 bacterial phyla identified are found in the lower GI tract [83]. Two of these phyla are totally dominating: *Firmicutes *(65%–80% of the clones) and *Bacteroidetes *(about 23%), while *Actinobacteria *(about 3%), *Proteobacteria *(1%) and *Verrumicrobia *(0.1%) exist only in modest amounts [76,84,85]. Of special interest is that *Actinobacteria* and *Firmicutes*, to which the genus *Lactobacillus* belongs, are almost exclusively Gram-positive, while *Bacteroidetes* and *Proteobacteria *are mainly Gram-negative [86]. Recent attempts to study the microbiota at other sites within the digestive tract report that the mouth harbors the greatest phylogenetic diversity, the stomach the lowest, and diversity to increase from stomach to the stool [87]. 

## 11. Personal Experience with Pro- and Synbiotics

My personal interest in microbiota and probiotics started in the early 1980s. Since 1963, I have been involved in the development of liver extensive surgery and active in the search for new tools to combat the unacceptably high rate of peri-operative infections, which was and still is associated with major surgery in general and in particular with extensive liver resections. At that time, it was standard practice to treat patients with an antibiotic umbrella for at least the first five post-operative days, in the belief that this treatment would reduce the rate of post-operative infections. However, a review of our last 81 liver resections gave unexpected information, which directed my interest to human microbiota and the possibility of using probiotics as an alternative infection prophylaxis. From this study, it was shown that only 57/81 patients had, in fact, received antibiotic treatment; this prophylaxis had been neglected in the remaining 24/81 patients [88,89]. It was surprising that there were no cases of sepsis in the group of patients who had not received prophylactic antibiotics with sepsis incidence confined to the antibiotic-treated patients. There was, at that time, a growing awareness of the importance of human microbiota [90] and to contemporaneous published studies that had attempted to recondition the gut through the supply of lactobacilli [91]. 

There was also at that time a growing understanding that not only disease, but also lifestyle and prescribed chemicals and pharmaceuticals, could impair microbiota immune defense. The use of probiotic treatment, as an alternative means of preventing unwanted infections in disease in general, but particularly in surgical and critically ill patients, appeared as an attractive option. This was the reason why I established collaborative efforts with experts in microbiology, chemistry, nutrition and experimental and clinical science to seek, develop and test probiotics both experimentally and clinically, which could be expected to constitute powerful tools to prevent sepsis of various kinds. 

Interdisciplinary collaboration in the early 1990s led to the identification of some *L. plantarum *strains that demonstrated strong anti-inflammatory capacities. *L. plantarum *299, later used together with oatmeal in a synbiotic composition [92,93,94], is produced and marketed by Probi AB, Lund, Sweden. I participated in this program until 1999, when I decided to re-direct my interest towards development and studies of a more complex synbiotic composition, designed not only to supplement four newly identified bioactive LABs in combination, but also four different prebiotic fibers, already known for their strong bioactivity. Our aim was to provide this composition in much larger doses than was the practice at that time. Furthermore, knowing that most of the important LABs rarely exist in the microbiota of Westerners encouraged us to seek potent probiotic bacteria normally growing on plants, instead of selecting bacteria normally found in human microbiota. 

Since 1999, all my efforts in this field have concentrated on a four LAB/four fiber composition, consisting of either a mixture of 4 × 10^1^^0^ (40 billion LAB, Standard version—Synbiotic 2000™) or a mixture of 10^11^ (400 billion Forte version—Synbiotic 2000 Forte™) based on the following four LAB: *Pediococcus pentosaceus* 5–33:3, *Leuconostoc mesenteroides* 32–77:1, *Lactobacillus paracasei* subsp. paracasei 19, and *Lactobacillus plantarum* 2362 in combination with 4 × 2.5 g of each of the following four fermentable fibers: betaglucan, inulin, pectin and resistant starch, in total 10 g of prebiotic fibers per dose [95,96], a formula that is currently a product produced by Synbiotic AB, Sweden. 

### 11.1. Perioperative Prophylaxis in Elective Surgery

*L. plantarum *299 in a dose of 10^9^ plus a total of 15 g of oat and inulin fibers was tried, under research condition, in patients undergoing extensive abdominal surgical operations. The patients were mainly derived from those undergoing liver, pancreatic and gastric resections, equally distributed between three groups and supplemented with either: (1) live LAB and fiber; (2) heat-inactivated LAB and fiber, and (3) standard enteral nutrition [97]. Each group comprised 30 patients. The 30-day sepsis rate was 10% (3/30 patients) in the two groups receiving either live or heat-inactivated LAB, compared to 30% (9/30 patients) in the group on standard enteral nutrition (*p* = 0.01) [97]. The largest difference was observed in the incidence of pneumonia: Group 1: 2 patients; Group 2: 1 patient; Group 3: 6 patients. The beneficial effects of treatment were seemingly most pronounced in gastric and pancreatic resections; the sepsis rate being: Group 1: 7%, Group 2: 17% and Group 3: 50%. The same pattern was observed for non-infectious complications: Group 1: 13% (4/30) Group 2: 17% (5/30); Group 3: 30% (9/30). The supply of antibiotics to Group 1 was significantly less (*p* = 0.04) than to the other two groups, with the mean length of antibiotic treatment also considerably shorter: Group 1: 4 ± 3.7 days; Group 2: 7 ± 5.2 days; Group 3: 8 ± 6.5 days. 

In a prospective, randomized, double-blind trial, 80 patients undergoing pylorus-preserving pancreatoduodenectomy (PPPD) received, twice daily, either Synbiotic 2000™ (2 × 40 billion LAB, *i.e.*, 80 billion LAB per day) or only the fibers in composition from the surgery the day before and during the first seven postoperative days [98]. A highly significant difference in infection rate (*p* = 0.005) was observed as only 5/40 patients (12.5%) in the Synbiotic 2000-treated group suffered infections (4 wounds and one urinary tract infection) *vs. *16/40 (40%) in the fiber-only group (6 wound infections, 5 peritonitis, 4 chest infections, 2 sepsis, and one of each of urinary tract infection, cholangitis and empyema)—Table 5. The number of infecting microorganisms were also statistically and significant reduced—see Table 6. Statistically significant differences between the groups were also observed regarding the use of antibiotics (mean: Synbiotic 2000, 2 ± 5 days; Only-fibers, 10 ± 14 days) [98].

**Table 5:** Infective complications in patients undergoing pancreatoduodenectomy [98]; *p* < 0.05.

	Synbiotic 2000	Fibers only
Wound infections	4	6
Urinary infection	1	1
Peritonitis	0	5
Pneumonia	0	4
Sepsis	0	2
Cholangitis	0	1
Empyema	0	1
	Total 5/40 (12.5%)	Total 16/40 (40%)

**Table 6:** Pathogens isolated from patients undergoing pancreatectomy treated with Synbiotic 2000 and only the fibers in Synbiotic 2000, respectively [98].

Isolated microorganisms	Synbiotic 2000	Fibers only
*Enterobacter cloacae*	2	8
*Enterococcus faecalis/faecium*	1	7
*Escherichia coli*	0	7
*Klebsiella pneumoniae*	2	2
*Proteus mirabilis*	1	1
*Staphylococcus aureus*	0	2
	Total 6	Total 27

In another randomized controlled study, 45 patients undergoing major surgery for abdominal cancer were divided into three treatment groups: (1) enteral nutrition (EN) supplemented with Synbiotic 2000 (LEN); (2) EN supplemented with only the fibers in the same amounts (20 g) (20 g) as in Synbiotic 2000™ (FEN); and (3) Standard parenteral nutrition (PN). All treatments lasted for two preoperative and seven days postoperative days. The incidence of postoperative bacterial infections was 47% with PN, 20% with FEN and 6.7% with LEN (*p* < 0.05). The numbers of infecting microorganisms were also statistically and significantly reduced—see Table 7. Significant improvements were also observed in prealbumin (LEN, FEN), C-reactive protein (LEN, FEN), serum cholesterol (LEN, FEN), white cell blood count (LEN), serum endotoxin (LEN, FEN) and IgA (LEN) [99].

**Table 7:** Pathogens recovered from patients undergoing surgery for abdominal cancer treated with Synbiotic 2000 and only the fibers in Synbiotic 2000, respectively [99].

Isolated microorganisms	Synbiotic 2000	Fibers only
*Pseudomonas aeruginosa*	17	24
*Staphylococcus aureus*	8	11
*Staphylococcus epidermidis*	1	1
*Staphylococcus faecalis*	-	1
*Enterobacter cloacae*	4	-
*Acinetobacter *spp.	2	3
*Staphylococcus haemolyticus*	-	1
*Serratia* spp.	-	2
*Klebsiella* spp.	-	1
*Proteus mirabilis*	-	2
*Candida albicans*	2	6
*Aspergillus *spp.	-	-
*Bacillus subtilis*	-	1
*Klebsiella* spp.	-	1
	Total 34	Total 54

### 11.2. Perioperative Prophylaxis in Liver Transplantation

A prospective, randomized study in 95 liver transplant patients supplemented *L. plantarum *299 in a dose of 10^9^ plus 15 g of oat and inulin fiber [100]. Three groups of patients were studied: (1) selective digestive tract decontamination (SDD) four times daily for six weeks; (2) *L. plantarum *299 (LLP) in a dose of 10^9^ plus 15 g of oat and inulin fibres supplied postoperatively for 12 days; and (3) identical to group 2 but with heat-killed *L. plantarum *299 (HLP). Identical enteral nutrition was supplied to all patients from the second postoperative day. The numbers of postoperative infections were SDD 23, LLP 4 and HLP 17. Signs of infections occurred in SDD 48% (15/32), in LLP 13% (4/31), *p* = 0.017 nd HLP 34% (11/32), respectively. The most dominant infections were cholangitis (which occurred: SDD 10, LLP in 2, and HLP in 8) and pneumonia (which occurred: SDD in 6, LLP in 1, and HLP in 4). There was a statistically significant reduction in the numbers of infecting microorganisms, the most frequently isolated microbes being *Enterococci* and *Staphylococci*. Patients requiring hemodialysis were SDD: 8; LLP: 2 and HLP: 4 and the number of re-operations SDD: 6; LLP: 4 and HLP: 2, respectively. There were no deaths. The stay in ICU, the hospital stay, and length of antibiotic therapy was shorter in the LLP group, but did not reach statistical significance. The CD4/CD8 ratio was also higher in the LLP group compared to the other two groups (*p* = 0.06). 

In a subsequent study, 66 human orthotopic liver transplant patients were randomized to either receive Synbiotic 2000 or only the fibers in Synbiotic 2000. The treatment was started on the day before surgery and continued for 14 days after surgery. During the first postoperative month, only one patient in the Synbiotic 2000-treated group (3%) showed signs of infection (urinary infection) compared to 17/33 (51%) patients in those supplemented with only the four fibers [101]. Only one infecting organism was cultivated in the Synbiotic-treated group, which was shown to be *Enterococcus fecalis*, in contrast to seventeen organisms in the fiber-only treated group—see Table 8. The use of antibiotics was on average 0.1 ± 0.1 day in the Synbiotic-treated patients and 3.8 ± 0.9 day in the fiber-only treated group [101].

**Table 8:** Pathogens isolated from patients undergoing liver transplantation treated with Synbiotic 2000 and only the fibers in Synbiotic 2000, respectively [101].

Isolated bacteria	Synbiotic 2000	Fibers only
*Enterococcus faecalis*	1	11
*Escherichia coli*	0	3
*Enterobacter cloacae*	0	2
*Pseudomonas aeruginosa*	0	2
*Staphylococcus aureus*	0	1
	Total 1	Total 18

### 11.3. Early Treatment in Major Trauma

Two prospective randomized trials with Synbiotic 2000™ and Synbiotic 2000 Forte™, respectively, were undertaken. In the first study [102], the patients were randomly allocated into four groups in order to compare the efficacy of preventing both total infections and, particularly, chest infections of the three commercial nutrition solutions: Group A: Alitraq (Abbott-Ross, Abbott Park, IL) 5.25 g protein, 16.5 g carbohydrate, 1.55 g fat and 1.55 g glutamine, 446 mg arginine, 154 mg α-linolenic acid per 100 mL. The Osmolarity is 480 mOsml/L. Group B: Nova Source (Novartis Medical Nutrition, Basel, Switzerland) 4.1 g protein, 14.4 g carbohydrate, 3.5 g fat, 2.2 g fermentable fibers as fermentable guar gum per 100 mL. The osmolarity is 228 mOsm/L. Group C: Nutricomp peptide (B. Braun, Melsungen, Germany) 4.5 g hydrolyzed protein, 16.8 g carbohydrate, 1.7 g fat per 100 mL. The osmolarity is 400 mOsm/L. A fourth solution, Nutricomp standard, was chosen to be tried in combination with Synbiotic 2000—Group D: Nutricomp standard (B. Braun, Melsungen, Germany) supplemented with Synbiotic 2000, Nutricomp standard contain 3.7 g protein, 13.7 g carbohydrate, 3.3 g fat per 100 mL. The osmolarity of this solution is 240 mOsm/L. One sachet of Synbiotic 2000 was added to the solution before delivery to the patients [102]. Nova Source and Nutricomp peptide did not reduce the levels of proinflammatory cytokines, while Alitraq and Nutricomp standard + Synbiotic 2000 significantly down-regulated Il-6, but not Il-8 and TNF-α. Table 9 lists the total number and number of chest infections associated with the various nutrition solutions. The group containing Synbiotic 2000 demonstrated significant reductions in both total infections as in chest infections; the total number of infections being reduced by about two thirds, and the number of chest infections being reduced to about half [102]—see Table 9. 

In the other study [103,104,105], 65 polytrauma patients were randomized to receive once daily, for 15 days following major trauma, either Synbiotic 2000 Forte (400 billion LAB + 10 g of fibers, see above) or maltodextrine, as placebo. Significant reductions were observed between the groups in the number of deaths (5/35 *vs.* 9/30, *p* < 0.02), severe sepsis (6/35 *vs*. 13/30, *p* < 0.02), chest infections (19/35 *vs.* 24/30, *p* < 0.03), central line infections (13/32 *vs.* 20/30, *p* < 0.02), and ventilation days (average 15 *vs*. 26 days [65]) [103,104,105]. A total of 54 pathogenic microorganisms were cultivated in the Synbiotic treated group compared to 103 in the maltodextrine group—see Table 10 [103,104,105]. Repeat analyses also revealed that serum levels of endotoxin (LPS) were decreased and “time to bloodstream infection” significantly prolonged in patients treated with Synbiotic 2000 Forte.

**Table 9:** Total number of infections and number of chest infections in severe trauma receiving four different commercial nutrition solutions of which one is combined with added Synbiotic 2000 [102].

	Total number of infections	Number of chest infections
Alitraq Abbott-Ross (glut + arg)	16/32 50%	11/32 34%
Nova Source Novartis (+guargum)	17/29 58%	12/29 41%
Nutricomp peptide Braun (+peptide)	13/26 50%	11/26 42%
Nutricomp standard (+Synbiotic 2000)	4/26 15%	5/26 19%

**Table 10:** Pathogens isolated from patients with polytrauma treated with Synbiotic 2000 and only the fibers in Synbiotic 2000, respectively [103,104,105].

Isolated microorganisms	Synbiotic 2000	Fibers only
*Acinetobacter baumanni*	21	35
*Candida albicans*	7	17
*Pseudomonas aeruginosa*	15	14
*Staphylococcus epidermidis*	2	10
*Staphylococcus aureus*	4	7
*Staphylococcus hominis*	-	2
*Enterobacter aerogenes*	-	2
*Staphylococcus haemolyticus*	-	1
*Serratia* spp.	-	2
*Klebsiella* spp.	5	12
*Proteus* spp.	-	1
	Total 54	Total 103

### 11.4. Early Treatment in Severe Acute Pancreatitis

In a further study, patients with severe acute pancreatitis were randomized to receive either a freeze-dried preparation containing live *L. plantarum *299 in a dose of 10^9^ together with a substrate of oat fiber or a similar preparation but heat-inactivated, administered daily through a nasojejunal tube for seven days [105]. The study was concluded when, on repeat statistical analysis, significant differences in favor of one of the two groups were obtained. This occurred when a total of 45 patients had entered the study. 22 patients had, at that time, received treatment with live, and 23 with the heat-killed, *L. plantarum *299. Infected pancreatic necrosis and abscesses were seen in 1/22 (4.5%) in the live LAB group *vs.* 7/23 (30%) in the heat-inactivated group (*p* = 0.023). The only patient in the lactobacillus group who developed infection, a urinary infection, did so on the fifteenth day, *i.e.*, at a time when he had not received treatment for eight days. The length of stay was also considerably shorter in the live LAB group (13.7 days *vs.* 21.4 days) but the limited size of the material did not allow the statistical analysis to reach full significance [106].

Sixty-two patients with severe acute pancreatitis (SAP) (Apache II scores: Synbiotic 2000-treated 11.7 ± 1.9, controls 10.4 ± 1.5) were given either two sachets/day of Synbiotic 2000™ (2 × 40 billion LAB/day and totally 20 g fibers) or the same amounts of fibers (20 g) as in Synbiotic 2000™ during the first 14 days after arrival at the hospital [106]*.* 9/33 patients (27%) in the Synbiotic 2000-treated group and 15/29 patients (52%) in the fiber-only treated group developed subsequent infections. 8/33 (24%) of the Synbiotic 2000-treated and 14/29 (48%) of the fiber-only treated patients developed SIRS, MOF or both (*p* < 0.005). A total of seven pathogenic microorganisms were cultivated in the Synbiotic-treated group compared to seventeen in the fiber-only group [107]—see Table 11.

**Table 11:** Pathogens isolated from patients with acute pancreatitis treated with Synbiotic 2000 *versus* those receiving only fibers [107].

Isolated microorganisms	Synbiotic 2000	Fibers only
*Pseudomonas aeruginosa*	1	4
*Enterococcus faecalis*	1	2
*Enterobacter* spp.	1	1
*Streptococcus* spp.	2	-
*Staphylococcus* *aureus*	1	1
*Enterococcus faecium*	1	-
*Candida* spp.	-	2
*Staphylococcus haemolyticus*	-	1
*Serratia* spp.	-	2
*Klebsiella* spp.	-	1
*Escherichia coli*	-	1
*Stenotrophomonas maltophilia*	-	1
*Citrobacter freundii*	-	1
	Total 7	Total 17

In a recently published third study [108], Synbiotic 2000 Forte was administered within 24–48 h of symptoms of sickness to patients with severe acute pancreatitis and compared to 32 patients on a control formula, the average volume and amount of calories being the same in the two treatment groups. The study demonstrated a lower infection rate (including pancreatic and peripancreatic necrosis); secondary infections (2 *vs.* 9, *p* = 0.0001), septicemia (2 *vs.* 7, *p* = 0.03), lower rate of surgical interventions, (3 *vs.* 12, *p* = 0.005), shorter stay in ICU (8 *vs.* 16 days *p* = 0.05), shorter hospital stay (23 *vs.* 36 days, *p* = 0.03) and reduced mortality (0 *vs.* 17 patients, *p* = 0.02) [108].

### 11.5. Effects on “Mind Clarity”—Encephalopathy

Patients with critical illness, as well as patients with chronic disorders such as liver cirrhosis and diabetes, frequently suffer a mild but sometimes severe confusion, which often has its origin in the gut [109]. Increasing evidence suggest that probiotics, alone but also in combination with plant antioxidants and fibers, possess strong neuro-endocrine modulatory effects and can alleviate the effects of physical and mental stressors [110,111]. We undertook some studies to explore the effects of Synbiotic in patients with liver cirrhosis and minimal encephalopathy (MHE) [112]. Fifty-five patients with MHE were randomized to receive for 30 days: (1) Synbiotic 2000 (*n* = 20); (2) the fibers in the composition alone (*n* = 20); or (3) a placebo (*n* = 15). All cirrhotic patients with MHE were found to have severe derangements of the gut micro-ecology and significant overgrowth of potentially pathogenic *Escherichia coli *and *Staphylococcal *species. Synbiotic treatment significantly increased the fecal content of non-urease-producing *Lactobacillus *species and reduced the numbers of potentially pathogenic micro-organisms. The treatment was also associated with a significant reduction in endotoxemia and in blood ammonia levels. A documented reversal of MHE was obtained in half of the treated patients, while the Child-Turcotte-Pugh functional class improved in about 50% of cases [112]. Treatment with fermentable fibers alone also demonstrated substantial benefits in a proportion of patients. 

In a second study, 30 cirrhotic patients were randomized to receive either Synbiotic 2000 or placebo for only seven days [113]. Viable fecal counts of Lactobacillus species, Child-Pugh class, plasma retention rate of indocyanine green (ICGR15), whole blood tumor necrosis factor alpha (TNF-a) mRNA and interleukin-6 (IL-6) mRNA, serum TNF-a, soluble TNF receptor (sTNFR)I, sTNFRII and IL-6 and plasma endotoxin levels were measured, pre- and post-treatment. The treatment with Synbiotic 2000 was associated with significantly increased fecal lactobacilli counts and significant improvements in ICGR15 and Child-Pugh class. Significant increases in whole blood TNF-a mRNA and IL-6 mRNA, along with serum levels of sTNFRI and sTNFRII, were also observed and TNF-a and IL-6 levels correlated significantly, both at baseline and post-Synbiotic treatment. Synbiotic-related improvement in ICGR15 was accompanied by significant changes in IL-6, both at mRNA and protein levels, but this was unrelated to levels of plasma endotoxin. No significant changes in any parameter were observed following placebo treatment. This study concluded that even short-term synbiotic treatment significantly modulated gut flora and improved liver function in patients with cirrhosis [113]. Minimal encephalopathy is common not only in liver cirrhosis, but is also seen in other chronic diseases such as diabetes. The observations in patients with liver cirrhosis gives hope that Synbiotic treatment may also be effective in other chronic diseases. See also [114].

### 11.6. Effects in HIV

It is well documented that disturbance of the microbiota occur early in HIV-1 infection, which leads to greater dominance of potential pathogens, reduced levels of Bifidobacteria and lactobacillus species and increasing mucosal inflammation. Current and emerging studies support the concept that probiotic bacteria can provide specific benefit in HIV-1 infection. It was not until Brenchley *et al. *in 2006 identified translocation of microbes or microbial products without overt bacteremia, as a major cause of systemic immune activation in HIV-1 and SIV infection [115], that a greater interest in bio-ecological treatment emerged.

Impairment of the GI tract in HIV-positive patients is already present in the early phases of HIV disease and is associated with elevated levels of intestinal inﬂammatory parameters and definite alterations in the gut commensal microbiota, confirming a possible correlation between intestinal microbial alteration, GI mucosal damage, and immune activation status, further confirming that alterations at the GI-tract level are a key factor in the pathogenesis of chronic HIV infection [116]. The findings, in a recent study, of fairly mild changes in microbiota of HIV-infected individuals, before initiation of pharmacological treatment, might suggest that the later-observed profound alterations in microbiota could be pharma-induced, as only a trend to a greater proportion of Enterobacteriales compared to control subjects (*p* = 0.099) were observed, despite the significant negative correlations between total bacterial load and duodenal CD4+ and CD8+ T-cell activation levels [117]. As pointed out in a recent review, current and emerging studies appear to support the concept that probiotic bacteria can provide specific benefit in HIV-1 infection. Probiotic bacteria have proven to be active against bacterial vaginosis in HIV-1 positive women and have enhanced growth in infants with congenital HIV-1 infection [118]. Probiotic bacteria may also stabilize CD4+ T-cell numbers in HIV-1 infected children and are likely to have protective effects against inflammation and chronic immune activation of the gastrointestinal immune system [118]. 

Recent studies at least partly support the assumption that *L. rhamnosis* GR-1 and *L. Reuteri* RC-14 tend to increase the probability of a normal vaginal flora (odds ratio 2.4; *P* = 0.1) and significantly increase the probability of a beneficial vaginal pH (odds ratio 3.8; *p* = 0.02) at follow-up [119,120]. However, later attempts using probiotic yoghurts have proven less successful [121]. In a recent pilot study of 38 women with HIV taking highly active antiretroviral therapy (HAART), Synbiotic 2000 Forte was supplemented orally for four weeks [122]. In a surprising and very encouraging observation, the supplemented formula showed the ability, despite heavy pharmaceutical treatment, to survive during the passage through the GI tract, and also the ability to colonize the gut and contribute to a significantly elevated level in the stool of the supplemented LAB group. The T-cell activation phenotype was altered by exposure to the Synbiotic formula and was accompanied by a slightly elevated HLA-DR expression of a minor population of CD4+ T-cells, which normally lack expression of HLA-DR or PD-1. These significant changes occurred in the context of unaltered microbial translocation, as measured by plasma bacterial 16S ribosomal DNA [122]. It is especially encouraging that the LAB supplemented with Synbiotic 2000, despite heavy medication/highly active antiretroviral therapy (HAART), were able to colonize the gut and seemingly, at least slightly, improve immune functions. Hopefully, significantly more pronounced positive effects will be obtained the day we are ready to try eco-biological treatment, not only as complementary treatment, but as an alternative to, pharmaceutical treatment.

## 12. It Is All about Inflammation

Inflammation, an essential component of immune-mediated protection against pathogens, tissue damage, and uncontrolled immune responses, will commonly, especially in Westerners, institute a state of chronic inflammation, which occurs when the immune response is activated despite absence of “danger” signals or fail to completely clear such signals. Numerous factors, in addition to genetic predisposition, trauma and various stress factors (physical and emotional) are known to contribute to increased discrete and long-lasting inflammation, among them age, diet and medications. 

Studies of human gene-related inflammation suggest that, of the approximately 25,000 human genes, approximately 5%, or some 1200 genes, are involved in inflammation [123,124,125]. It is increasingly understood that the human genome in itself will only explain a minority of chronic diseases, far less than changes in lifestyle, food habits and social behavior, factors which seem to have a dominating impact on human health. Clearly, the molecular mechanisms linking environmental factors and genetic susceptibility was first envisioned after the recent exploration of the, until recently, hidden source of genomic diversity, *i.e.*, the metagenome with its more than 3 million genes [126]. Although the mechanisms behind the metagenome-associated low-grade inflammation and the corresponding immune response are yet not fully understood, there is no doubt that the metagenome has a dominating influence on altered body functions such as adipose tissue plasticity and metabolic syndrome-associated diseases such as hepatic steatosis, insulin resistance and cardiovascular diseases, but also on, for example, autoimmune diseases such as rheumatoid arthritis, gastrointestinal and neuropsychiatric diseases, on the development and progress of cancers [127], and many other chronic disorders. When disease exacerbations occur, in trauma or in critical illness, the normally silent or discrete inflammation turn into a “storm” [128] as experienced in systemic inflammatory response syndrome (SIRS) and multiple organ failure (MOF) [129]. In many severe conditions like MOF and SIRS, components of cytokine-induced injury might be more damaging than the initial cause/trauma/early invasion of micro-organisms. Inflammatory cytokines, such as TNF alpha and IL-1β, released by these events will destabilize endothelial cell–cell interactions and cripple vascular barrier function, producing capillary leakage, tissue edema, organ failure, and often leading to death [129].

## 13. Special Focus on Fat Metabolism in Liver and Pancreas Disease

Modern humans have much more fats in the abdomen than our paleolithic forebears. The amount of fat in the abdomen can vary from a few milliliters in a lean subject, to approximately 6 L in gross obesity [130]; abnormal amounts of abdominal fat accumulation were not possible before the advent of modern agriculture. Visceral adipocytes are, compared with subcutaneous fat cells, known to secrete many more free fatty acids, but also approximately three times as many of pro-inflammatory molecules such as IL-6 and PAI-1 per gram tissue [131]. These observations explain not only the significantly higher risk of developing various chronic but also acute diseases and complications to invasive treatments in individuals with visceral obesity. The stress-induced load of mobilized fats and pro-inflammatory and pro-coagulant molecules from visceral adipocytes via the portal vein on the liver is much more than the organ can handle, especially when functionally reduced by disease and/or surgery—a load that often is increased up to 1000 times [131]. Other organs—the brain, the lungs, the pancreas and the kidneys—are also severely affected by this flood of pro-inflammatory factors. This development is serious in conditions where these organs are reduced in mass or functions, as in extensive liver resection, liver transplantation, as well as in patients with pre-existing reduced liver functions or suffering from conditions such as hepatic steatosis or liver cirrhosis. As a matter of fact, the accumulation of fat in the liver is dangerously heavy even if extensive liver resections are undertaken in young and healthy rats, being even more dramatic in humans [132,133]. The increase of hepatic fat remains often for at least one month and is associated with a corresponding reduction also in circulatory fats. It is advisable to strictly limit supply of nutritional fat to patients as long as this condition remains. 

Fatty livers are almost always associated with dysbiosis [134]. It is promising that steatosis of the liver can be significantly reduced by so-called eco-biological nutrition, plant-derived antioxidants and nutrients [68,69,135,136], and particularly by supply of efficient probiotics [68,69,137]. Recent experimental studies report significant hepato-protective effects of turmeric-derived curcumin; stabilization of redox state, reduced liberation of liver enzymes, and attenuated expression of pro-inflammatory cytokines, reduced infiltration of fat in the liver after liver resection [138] and after toxic injuries [139]. The present Western lifestyle and consumption of high-calorie-loaded Western foods is responsible for the high incidence of chronic hepatic steatosis - every fourth Westerner claims to suffer from this condition.

## 14. Inflammation Control—Pharma and/or Probiotics?

Inflammation is, as discussed above, extraordinarily complex. In rheumatoid arthritis (RA), for example, the joints are rich in cytokine-secreting cells containing a wide range of effector molecules including pro-inflammatory cytokines such as IL-1β, IL-6, TNF-α and IL-18, chemokines such as IL-8, IP-10, MCP-1, MIP-1 and RANTES, MMPs such as MMP-1, -3, -9 and -13 and metabolic proteins such as Cox-1, Cox-2 and iNOS, which interact with one another in a complex manner suggested to lead to a vicious cycle of high pro-inflammatory signals and chronic and persistent inflammation [140,141]. NF-КB is suggested to be the master regulator of inflammatory cytokine production in RA. Almost identical mediators are involved in other autoimmune disorders, such as inflammatory bowel diseases [142]. This knowledge has led to development to a new generation of pharmaceutical drugs, generally referred to as biological, designed to inhibit the crucial mediators of pro-inflammatory signals and the subsequent abnormal immune response. A whole series of revolutionary new drugs such as anti-TNF-α, anti-IL-1β, anti-HER2, *etc.* are already successfully tried, and new drugs such as antibodies targeting IL-12/IL-23 pathways, IFN-γ, IL-17A, IL-2 and IL-6, and inhibitors of NF-КB are also tried in a variety of chronic inflammatory and autoimmune diseases. Some of these have already demonstrated initially promising results, while other treatments, such as administration of the regulatory cytokines IL-10 and IL-11, have failed to induce reproducible clinical effects [142]. Significant benefits in quality of life and in tissue/organ healing are encountered in at least more than 50% of treated patients. These drugs are also in most cases tolerated well, but adverse events such as infections such as reactivating tuberculosis, tumors such as lymphomas, and demyelinating diseases, are sometimes described in patients treated with these drugs. Such consequences are acceptable as long as these drugs are used in diseases that are refractory to all other treatments, but this may be an issue when, as increasingly suggested, they are tried in early stages of diseases, similar to what has happened with the indications for statins [143].

## 15. Single or Multiple Gene Targeting as Treatment?

Most modern pharmaceuticals made to control inflammation, often referred to as biologicals, are designed to target single genes among those regarded as responsible for the etiology of disease, even if these drugs, in reality, will affect several other genes, as well. Sometimes the results of selective targeting may be short-lasting, and increased inflammation will sooner or later come back, allowing the inflammatory process to find new pathways and the disease to consequently continue its progress.

**Figure 2:**
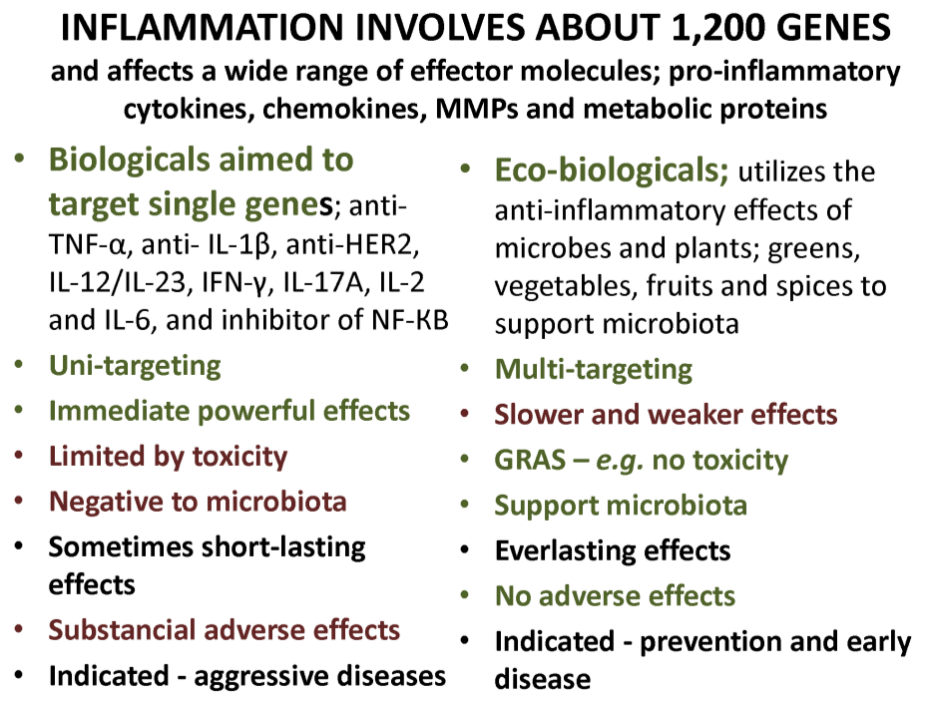
Molecular gene targeting to prevent or reduce severe systemic inflammation, development and cure of chronic diseases—effects of biological and eco-biological treatment options.

It is unfortunate that no studies thus far have addressed the effects of the biologicals on microbiota and leaking barriers. Until such studies have been conducted, one must assume that these drugs have the same devastating effects on microbiota and barrier function similar to, or worse than, those of other drugs. Plant-derived mediators, or phytochemicals, such as curcumin, resveratrol, genistein, *etc.* (see above) and plant fibers, particularly prebiotic fibers, and probiotic bacteria, which may be termed “eco-biologicals” possess, alone or in combination, the same molecular abilities as biologicals—although much weaker—but also without any adverse effects. These compounds are all classified as GRAS, generally considered as safe, and should be considered especially when the main indication is prevention, is early in the disease and, especially, as palliative treatment in children and the elderly—Figure 2.

## 16. Asian Foods Associated with Less Disease

**Figure 3:**
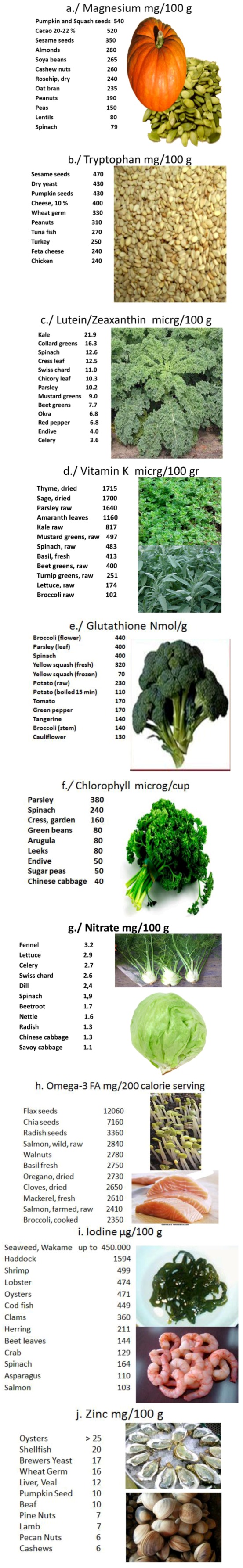
Examples of foods rich in key nutrients, which might be tried in future nutrition solutions both for healthy and critically ill individuals.

Several population-based studies indicate that people in Southeast Asian countries have a much lower risk of developing colon, gastrointestinal, prostate, breast, and other cancers than their Western counterparts. They also demonstrate significantly reduced incidence, at least when living under rural conditions, of other chronic diseases such as coronary heart diseases, neuro-degenerative diseases, diabetes, inflammatory bowel diseases, *etc.* A significantly lower morbidity and mortality is often observed in countries in this part of the world. Morbidity and mortality in critical illness might also be significantly reduced in Southeast Asian countries, especially when compared to the West. It is likely that the frequent use in these countries of dietary constituents containing antioxidant/chemo-preventive molecules (see Figure 3), such as garlic, ginger, soybeans, turmeric, onion, tomatoes, cruciferous vegetables, chilies, and green tea, and many others, may play an important role not only in protection from chronic diseases, but also the poor outcome in critical care, as these dietary agents have documented ability to suppress transformative, hyper-proliferative, and inflammatory processes [144].

The present knowledge, as obtained from extensive research, especially in recent years, suggests that good health and well-being, in addition to regular physical exercise, good sleep and control of stress/spiritual harmony is strongly associated with the food we eat, and its influence on bodily functions, particularly microbiota. It is unfortunate that the sickest patients are more or less in constant stress, cannot exercise, and receive the worst nutrition. The modification of these conditions should be a highly prioritized challenge. 

## 17. Seven NOs and Three YESes!

I suggested, in a recent review, ten important principles in striving for optimal nutrition [78]:

- Restrict intake of insulinogenic, IGF1-rich or IGF1-stimulatory and Toll-stimulatory foods, such as refined carbohydrates: cereals, bread, sweets, cookies, rice, pasta, cooked tubers, including potatoes—these are foods which are absorbed high up in the small intestine and are of minimal benefit to microbiota.
- Restrict daily intake of fructose to below 25 grams a day.
- Restrict intake of dairy products, especially butter, cheese and milk powder, rich in saturated fats, hormones and growth factors such as IGF1, and also meat intake, especially inflammation-inducing, processed and cured meat such as bacon and sausages. See also [145,146,147].
- Restrict/eliminate intake of foods heated above 100 °C, known to be rich in the inflammation-inducing molecules AGEs and ALEs, and particularly foods heated above 130 °C, as foods with increased temperature become increasingly rich in pro-inflammatory and carcinogenic substances such as acrylamide and heterocyclic amines, e.g., fried and grilled foods, and toasted and high-temperature baked breads. See also [145,146,147].
- Restrict exposure to microbe-derived high inflammation-inducing endotoxins, especially found in meat hung for several days, hard cheeses, pork and ice creams.
- Restrict, eventually eliminate, intake of foods rich in proteotoxins such as casein, gluten and zein.
- Restrict intake of chemicals, including pharmaceutical drugs, to only what is absolutely necessary, as most chemicals are likely to be detrimental to microbiota.
- Increase dramatically the intake of fresh and raw greens, fresh spices and vegetables, rich in antioxidants, fibers, minerals and nutrients, but also inflammation-controlling factors such as curcumin, resveratrol and many others—see Figure 3.
- Increase/favor intake of ancient antioxidant-rich, high-fiber, low-calorie-containing grains such as buckwheat, amaranth, chia, lupin, millet, quinoa, sorghum, taro, teff, *etc*., and also the intake of beans, peas, chickpeas, lentils, nuts and almonds. See Table 12.
- Supplement large doses of vitamin D and omega fatty acids, both of special importance for control of inflammation and for function of microbiota. If ill—also supplement pro-/synbiotics, but only brands with documented clinical effects.

## 18. Nutrition of the Sick Made to Mimic Healthy Foods for the Healthy

If supplies of health-supporting nutrients are important to already healthy individuals, there is no doubt that it is even more important for ill patients. It is increasingly recognized that the artificial foods available today, whether made as pet foods, baby formulas or clinical nutrition solutions, often contribute to the development or aggravation of disease. It is especially so with the clinical nutrition solutions presently on the market, often produced from cheap raw materials such as evaporated milk or milk powder, and are not made to mimic natural healthy foods. Patients are not all the same, and they have different needs. Consequently, requests are made for specific formulations for conditions such as diabetes [148] and obesity [149]. Currently, there are approximately 230 different enteral feeding formulas and nutrition supplements commercially available [149], though there should be a place for more specialized “adapted-to-need” formulas.

## 19. Source of Protein Is Critical

The protein needed in the formula can be obtained from a large variety of sources, animal proteins, as well as plant proteins. Animal proteins commonly used in clinical nutrition formulas are usually milk based and consist either of casein (80% of milk proteins), whey (20% of milk proteins), or a mixture of both. Caseins are colloidal aggregations of as1-, as2-, b- and k-, while whey comprises fractions of β-lactoglobulin, α-lactalbumin, lactoferrin, various immunoglobulins, proteose-peptone and serum albumin. All proteins, irrespective of source, seem to be effectively metabolized in the small intestine. A recent study in healthy humans suggests that proteins that pass the terminal ileum are already at that stage dominated by bacterial protein; ~60% bacterial protein, ~15% mucin protein, ~7% soluble-free protein, and ~5% protein from intact mucosal cells, indicating a substantial microbial activity already within the human distal small intestine [150]. 

Limited data seems to be available to specifically support the choice of these protein sources. The biological effects vary between different types of proteins. Some proteins, such as Alpha-lactalbumin, gelatin and gelatin fortified with tryptophan, are reported to provide a higher degree of satiety (approx. 40%) than any of the commonly used proteins in nutrition formulas—casein, soy, whey, whey fortified with glycomacropeptide (GMP), for example—thus inducing a a significant reduction in energy intake (approx. 20%) [151]. Varying types of proteins are also absorbed differently and will consequently influence insulin response: Significant differences between fast-absorbing whey and soy and slow-absorbing casein have been observed [152].

## 20. Muscle Protein Synthesis Varies with Type of Protein Supplied

Enteral nutrients (EN) are known to potentiate the action of glucagon-like peptide-2 (GLP-2), a nutrient-regulated intestinotrophic hormone, derived from proglucagon in the distal intestine. When the ability of different proteins—casein, hydrolyzed soy, whey protein concentrate (WPC), and combined hydrolyzed WPC and casein—were tried in an animal study, only whey protein demonstrated the potential to enhance the ability of GLP-2 to reverse mucosal hypoplasia and increase mucosal cellularity, as well as the absorptive surface area [153]. Emerging evidence support that consumption of different types of proteins will have different stimulatory effects on the amplitude, and possibly also duration, of muscle protein synthesis after feeding, being greater after whey or soy protein consumption than after casein, both when measured at rest and after exercise [154].

The capacity of the proteins to inhibit systemic inflammation is of particular importance. When in an animal study the ability of three different protein sources to resist LPS-induced systemic inflammation was studied, a whey-based formula (Peptaman AF, Nestlé Healthcare Nutrition) was demonstrated as significantly superior to a commercial casein-based diet (Promote^®^ Abbott) and a standard soy-based diet high in cysteine and crude fiber [49]. Another animal study demonstrated that the supply of whey protein to exercise-trained rats led to, in comparison to casein-supplied and soy-supplied animals, significantly higher levels of liver glycogen [155]. Furthermore, a recent animal study suggests that whey proteins might possess decidely stronger anti-inflammatory abilities and to significantly decrease levels of IL-1β, TNF-α, IL-6, IL-4, malondialdehyde (MDA), nitric oxide (NO), reactive oxygen species (ROS), and neutrophil infiltration, while reducing wound healing time [156]. 

## 21. Plant-Based Proteins May Have Advantages

The use of whey proteins seems to have benefits, particularly over casein-based proteins, and most likely also over soy-derived proteins. It is unfortunate that similar information from human studies are largely lacking, despite widespread clinical use of milk-based formulations. However, a recent study in young boys fed casein demonstrated a significant 15% (*P* < 0.0001) increase in pro-inflammatory IGF1/s, but no changes in fasting insulin (*P* = 0.36), while boys fed whey instead had a 21% (*P* = 0.006) increase in fasting insulin, and no change in IGF-1 (*P* = 0.27) [157]. A critical review examining twenty-five recently published intervention trials examining chronic and/or acute effects of whey protein supplementation on lipid and glucose metabolism, blood pressure, vascular function and on the musculoskeletal system, concluded that although whey protein may affect glucose metabolism and muscle protein synthesis, the evidence for its clinical efficacy is not strong enough to make final recommendations for its use [158]. 

Plant-based proteins might have advantages. A recent study [159] demonstrated a great potential of feeding rice protein, especially as it seems to possess the strong ability to improve oxidative stress through enzymatic and non-enzymatic antioxidative defense mechanisms; the total antioxidative capacity (T-AOC), mRNA levels of glutamate cysteine ligase catalytic subunit (GCLC) and glutamate cysteine ligase modulatory subunit (GCLM) mRNA levels, antioxidative enzyme activities (T-SOD and CAT) and glutathione metabolism related enzyme activities. γ-glutamylcysteine synthetase (γ-GCS), glutathione S-transferase (GST), glutathione reductase (GR) and glutathione peroxidase (GSHPx) were all effectively stimulated by feeding rice protein when compared to feeding casein. Feeding rice protein did significantly reduce the hepatic levels of markers of inflammation such as malondialdehyde (MDA) and protein carbonyl (PCO) [159].

**Table 12:** Chemical composition of amaranth, quinoa, buckwheat in comparison to wheat seeds [159].

Seed	Protein	Fat	Total starch	Dietary fiber
Amaranth	16.5	5.7	61.5	20.6
Quinoa	14.5	5.2	64.2	14.2
Buckwheat	12.5	2.1	58.9	29.5
Wheat	12.0	2.5	63.0	17.4

## 22. Pseudocereals—A Promising Alternative Protein Source

So-called pseudocereals—amaranth, quinoa and buckwheat, to name a few examples—are known to have a somewhat higher content of protein than, for instance, wheat. They decidedly have a higher fat content (amaranth and quinoa) and also a favorable content of dietary fibers (especially amaranth and buckwheat), thereby possibly representing promising alternatives for the production of clinical nutrition formulas—see Table 12 [160]. The composition of fat in amaranth, quinoa, buckwheat and wheat seeds is similarly favorable with their high content of oleic acid (C18:1): amaranth 23.7; quinoa 26.7; buckwheat 33.6; wheat 13.2. For monounsaturated fats: amaranth 23.9; quinoa 28.1; buckwheat 34.7; and wheat 13.4—all g/100 g. 

Cereals, pseudocereals, and leguminous flours are all known to have a high content of amino acids [160], and an amino acid pattern much similar to the human muscle—especially buckwheat—and to constitute excellent substrates for the biosynthesis of γ-aminobutyric acid (GABA), which is known to effectively reduce inflammation and prevent tissue injury [161], but also to have strong “calming effects,” effective against fear, anxiety, depression, headaches and other mental conditions [162]. Strains of *Lactobacillus plantarum* and *Lactococcus lactis* have been proven effective to ferment cereals, pseudocereals, and leguminous flours and effectively release amino acids and GABA. A blend of buckwheat, amaranth, chickpea and quinoa flours in the proportions 1:1:5.3:1, when fermented with *L. plantarum C48*, was recently demonstrated to produce a high concentration of free amino acids (*ca.* 4467 mg/kg) and GABA (504 mg/kg) [163,164]. It is my opinion that, in the future, it should be possible to use a similar technology to produce new, hopefully more efficient clinical nutrition formulations.

## 23. Source of Fats for Clinical Nutrition

Mainly three other fat emulsions, apart from the commonly used soybean oil (SO)-based emulsions, have been used in parenteral nutrition formulas: medium-chain triglycerides (MCTs), olive oils (OOs), and fish oil (FOs). Enteral nutrition solutions have, to a large extent, been done to mimic parenteral nutrition solutions. It is most likely, as different oils are metabolized via different pathways, that various fats may affect different functions in the body; they may either induce or inhibit inflammation in the body or reduce or enhance immune suppression. A recent small study compared 12 patients receiving a mixture of soybean and medium-chain triglyceride oils (SMO) with 18 patients receiving a fat emulsion with part of the lipid replaced by fish oil, consequently reporting a trend toward reduced serum inflammatory cytokines in the fish-oil group as significant differences in interleukin (IL)-1, IL-8, and interferon (IFN)-γ was observed on postoperative day 4 (*P* < 0.05) and IL-1, IL-8, IFN-γ, IL-6, and tumor necrosis factor-α on postoperative day 7 (*P* < 0.05) [165]. Although not statistically significant, a reduction in postoperative liver dysfunction (fish-oil group *vs.* SMO group: 33% *vs.* 50%) and infection rate (fish-oil group *vs.* SMO group: 28% *vs. *42%) [165] was observed. However, other similar recent studies have found no significant difference between such groups [166]. 

## 24. Omega-3-Based Emulsions Receive Increasing Interest

Lipid mediators derived from the *n*-3 fatty acids eicosapentaenoic acid (EPA) or docosahexaenoic acid (DHA) are increasingly used in clinical nutrition to reduce systemic inflammation. Intercellular mediators such as protectins and resolvins, known to either protect from, or induce resolution of, inflammation, are generated from *n*-3 fatty acids in the body [167]. Experimental data suggest that *n*-3 fatty acids may improve acute lung injury and sepsis. Application of *n*-3 fatty acids to patients undergoing major surgery seems to provide some, although not dramatically different, beneficial effects—reduction of length of stay and decrease of infectious complications [168,169]. Recent human studies are, however, seemingly unable to support such conclusions, at least for critically ill patients on mechanical ventilation. A recent multicenter study [170] was undertaken involving 272 adults provided within 48 h of developing acute lung injury and requiring mechanical ventilation enteral supplementation of *n*-3 fatty acids, γ-linolenic acid, and antioxidants. The patients receiving the *n*-3 supplement demonstrated, compared to iso-caloric controls, an eight-fold increase in plasma eicosapentaenoic acid levels, more ventilator-free days (14.0 *vs.* 17.2; *P* = 0.02) (difference, −3.2 (95% CI, −5.8 to −0.7)), more intensive care unit-free days (14.0 *vs.* 16.7; *P* = 0.04), and fewer non-pulmonary organ failure-free days (12.3 *vs.* 15.5; *P* = 0.02). However, the sixty-day hospital mortality was 26.6% in the *n*-3 group *vs*. 16.3% in the control group (*p* = 0.054), and adjusted 60-day mortality was in the *n*-3 and control groups, respectively 25.1% and 17.6% (*P* = 0.11). Use of the *n*-3 supplement did also result in more days with diarrhea (29% *vs.* 21%; *P* = 0.001) [170].

Sixty-four adult patients undergoing surgery for gastrointestinal diseases were randomly assigned to receive isocaloric and isonitrogenous total parenteral nutrition with either an ω-3 fatty acid-enriched emulsion (Lipoplus, *n* = 32) or medium-chain triacylglycerols/long-chain triacylglycerols (Lipofundin; *n* = 32) for 5 days after surgery [171]. Total bilirubin decreased faster in the ω-3 fatty acid-enriched group (*p* = 0.017), and the so-called activated partial thromboplastin time was significantly prolonged between days 1 to 3 (*p* = 0.002) than in the other group. Although no differences were observed in C-reactive protein, interleukin (IL)-1, IL-8, IL-10, vascular endothelial growth factor (VEGF), and distribution of the T-cell subpopulation between the two groups, significant decreases in IL-6, tumor necrosis factor-α, and nuclear factor-κB, occurred in parallel to significant increases in leukotriene B5/leukotriene B4 [171]. 

## 25. Olive Oil-Enriched Emulsions May Have Benefits

Other plant-based sources of fat for clinical nutrition have also been tried. A recent study [172] compared parenteral administration to critically ill adults of an olive oil-based lipid emulsion with a standard soybean oil-based lipid emulsion, and found no differences in: rates of infectious and noninfectious complications, glycemic control, inflammatory and oxidative stress markers, and immune function. However, another recent prospective, randomized, controlled crossover study done by the same group and focusing on vascular, metabolic, immune, and inflammatory effects of 24-h parenteral infusion of an olive oil-based emulsion (ClinOleic) reported definite advantages of the olive-oil based formula compared to a soybean oil-based lipid emulsion (Intralipid); the soybean oil-based lipid emulsion increased blood pressure and impaired endothelial function—no such changes were observed with the use olive oil-based lipid emulsion or lipid-free enteral nutrition solutions [173].

## 26. A Great Need for New Enteral Nutrition Formulas

The shift in clinical praxis from an almost total parenteral nutrition praxis to an enteral nutrition-dominated praxis in post-surgical, post-trauma and critically ill patients has changed dramatically in clinical praxis and contributed to significant improvement in clinical outcome. However, the formulas for enteral nutrition have not changed to the extent that could have been expected; they continue to look much as they do when constructed for parenteral use. Although the knowledge about healthy and unhealthy foods has increased dramatically, and eating habits at least among health-concerned individuals have changed considerably, this information has not translated into a new generation of enteral nutrition formulas made to mimic natural healthy foods: rich in greens, never heated to high temperatures (low content of AGE and ALE), with no ingredients of proteotoxins such as gluten, casein and zein, *etc.* The content of plant-based foods—especially green leaves—match human dietary needs closer than other foods, and increasing evidence suggests this will lead to improved immune functions, increased resistance to disease and profoundly stronger tissues, especially muscles and tendons. There are thousands of varieties of greens and plant foods and each one has its own unique set of nutrients, which is why it is important to eat a variety of foods. Green smoothies rich in raw foods, greens (liquidized salads), avocadoes, bananas, carrots, fruits, *etc.*, as well as fresh raw-food based soups like the Spanish gazpacho, could constitute interesting alternatives, if hygienic conditions could be solved. 

## 27. Avocado: A Unique Fruit Rich in Healthy Oils, but Also Antioxidants

The increased interest in recent years in olive oil as a source of healthy fats is accompanied by interest in other plant-based sources of fats, such as avocado, coconut and red palm tree fruits. The last two had been demonized in the past due to their high content of saturated fat, but today, they are increasingly recognized for their health-promoting ingredients and clinical effects. The avocado fruit are especially rich in monosaturated fats, vitamins E and C, as well as carotenoids and sterols, important ingredients that possess antioxidant and radical scavenging activities often deficient in people in Western societies. Avocado is also particularly rich in lutein, alpha-carotene, beta-carotene, neoxanthin, violaxanthin, zeaxanthin, antheraxanthin, chlorophylls, and pheophytins. Supply of avocado or avocado oil has been shown to increase the uptake of carotenoids several-fold; lutein by 5 times, alpha-carotene 7 times, and beta-carotene 15 times [174]. Avocado oil has also been shown to reduce inflammation and protect tissues from destruction, especially observed on the musculoskeletal system [175]. 

## 28. Red Palm Oil: Rich in Vitamin A, MUFAs and Unsaturated Fats

The lipid profile of palm oil has a near 1:1 ratio of saturated to unsaturated fatty acids. It is regarded as the richest natural source of dietary pro-vitamin A carotenes, said to contain 15 times more pro-vitamin A carotenes than carrots and 300 times more than tomatoes; one teaspoon of red palm oil per day is enough to supply the recommended daily allowance (RDA) of vitamin A for children [176]. It is also a most abundant natural source of vitamin E, α-tocotrienol, and α-iocotrienol, lycopenes, squalene, Co-enzyme Q10, and saturated and unsaturated fatty acids (known to maximize absorption of carotenoid anti-oxidants). It is reported to possess unique tissue-protective capacity and especially strong neuro-protective properties. Palm oil-derived α-tocotrienol is said to reach the brain in sufficient quantity to attenuate stroke-mediated neuropathy [177]. Red palm oil with its high level of saturated fatty acid seems not, as feared earlier, to promote atherosclerosis and/or arterial thrombosis. The reason could be that most of the saturated fat in palm oil is medium-chain fatty acids (MCFA), which is absorbed directly into the portal vein, and rapidly transported to the liver for beta-oxidation, while long-chain fatty acids (LCFA), in the form of triacylglycerols, which dominate Western-type foods, are absorbed via the intestinal lymphatic ducts and transported by chylomicrons through the thoracic duct and directly into the systemic circulation, leading to a difference that could explain the dramatically different effects on systemic inflammation and health [178].

## 29. Red Palm Oil (and Coconut Oil): Effective against Inflammation

As a matter of fact, red palm oil has been reported to reduce the risk of arterial thrombosis and/or atherosclerosis, to inhibit endogenous cholesterol biosynthesis, platelet aggregation, reduce oxidative stress and significantly reduce blood pressure. It is suggested that dietary red palm oil, consumed in moderation by animals and humans, have the ability to promote efficient utilization of nutrients, activate hepatic metabolism of drugs, facilitate hemoglobinization of red blood cells and improve immune function [176]. In large parts of Africa, red palm oil is also regarded as potent inhibitor of the progress of HIV, but studies are lacking to support this belief. A recent *in vitro *study on human monocytic cells confirm that red palm oil has a remarkable ability to effectively inhibit LPS-induced generation of NO, production of PGE2, stimulation of secretion of pro-inflammatory cytokines (TNF-a, IL-4, and IL-8), and expression of iNOS, COX-2, and NF-jB [179]. The production of PGE2 and down-regulating of the expression of COX-2 and iNOS occurred in a dose-dependent manner, observations that support the potent anti-inflammatory activity of red palm oil with blockage of NF-КB activation and selective inhibition of COX-2 expression [180]. Studies demonstrating the ability of red palm oil to protect against ischemia and reperfusion injuries of the heart is most promising [180,181,182] and merits further investigations.

## 30. Medium-Chain Fatty Acids Have Anti-Infectious Effects and Do Not Produce Resistance!

Plant oil such as coconut or palm oil are rich in capric acid (C10:0) and lauric acid (C12:0), as well as the essential fatty acid, linoleic acid (C18:2). Capric acid (C10:0) and lauric acid (C12:0), also found in safflower oil, evening primrose oil and poppy-seed oil, are all recognized for their powerful bactericidal effects on a large number of bacteria, viruses and funguses. This information supports the specific role of these lipids on innate immunity. Specific fatty acids, especially those associated with phospholipids and particularly sphingolipids, are deeply involved in the physical barriers, permeability barriers, and immunologic barrier functions both of the skin and mucosal surfaces. The antibacterial actions of free fatty acids (FFAs) are typically broad spectrum and of potencies comparable to natural antimicrobial peptides (AMPs). While FFAs are not as structurally diverse as the more widely studied AMPs, their importance in the human innate immune system is well established, particularly in the defense of skin and mucosal surfaces [183]. Furthermore, the fact that FFA-resistant phenotypes as seen with conventional antibiotics rarely exist with antimicrobial fatty acids, makes plant-derived FFAs especially attractive for use both in medicine and in clinical nutrition. 

## 31. Urgent Need for New Eco-Biological Formulas

Disciplines like immunotoxicology and immunopharmacology are still in their infancy. Much evidence exists to support that the drugs we often use in ICUs have strong and hitherto sometimes unrecognized and often ignored negative influences on the immune system and to resistance against inflammation and infection [184]. The increasing information about the damage that most pharmaceuticals produce on microbiota renders it urgent for priority to be given to choice and use of pharmaceuticals, especially for the critically ill. It remains a great dilemma and a completely unsolved issue that the sickest and most demanding patients are not only under constant mental and physical stress with no chance of physical exercise, but also receive the most incomplete—even dangerous—nutrition, often containing ingredients documented to escalate inflammation and contribute to increased morbidity. 

There is an urgent need for a completely new set of enteral nutrition formulas. The formulas presently used are only slightly different compared to the artificial formulas used for parenteral nutrition, made mainly to provide calories and to support a favorable nitrogen balance. New insights necessitate formulas made mainly or entirely with the goal of restoring homeostasis in inflammation and immune functions. The new science of nutrigenomics provides tools to identify the effects of various food ingredients and their effects on various genes, particularly those associated with inflammation and immune functions. Immediate attempts should be made to avoid nutrition formula ingredients such as long-chain saturated fats, trans-fatty acids, advanced glycation end products (AGEs) [145,146,147], hormones, and various stress molecules and sugars, particularly fructose. Future enteral nutrition formulas should be made to mimic normal food as closely as possible. Certainly, some already-used regular foods, such as Mediterranean raw soups like the Spanish gazpacho and various other vegetable and fish soups, can be adapted for clinical use and provided, if necessary also by tube-feeding. Green leaves, fresh vegetable and fruit juices/smoothies can easily be adapted for clinical use and probiotic bacteria, prebiotic fibers, and plant antioxidants, various polyphenols, such as curcumenoids and resveratrol and similar molecules, can be mixed into these solutions. I have only recently realized the special importance of green leaves, especially when compared to roots; they have a low content of energy, but are enormously richn in important nutrients, minerals, vitamins, antioxidants—some being hundreds or thousand times richer in the leaves than in the roots—see Table 13 [185]. 

**Table 13:** Differences in ingredients between greens and roots of the same plant [184].

Nutrients	Beets 100 g	Beet greens 100 g Difference %		
Calories	43.00		22.00	−51
Protein (g)	1.61		2.20	+35
Fat total (g)	0.17		0.13	−76
Carbohydrate (g)	9.56		4.33	−45
Fiber Total (g)	2.80		3.70	+32
Sugar Total (g)	6.76		0.50	−740
Calcium (mg)	16.00		117.00	+731
Iron (mg)	0.80		2.57	+321
Magnesium (mg)	23.00		70.00	+304
Potassium (mg)	325.00		762.00	+235
Sodium (mg)	78.00		226.00	+290
Copper (mg)	0.08		0.19	+238
Selenium (mg)	0.70		0.90	+29
Vitamin C (mg)	4.90		30.00	+500
Riboflavin (mg)	0.04		0.22	+612
Vitamin A (IU)	33.00		6326.00	+19,169
Vitamin E (mg)	0.04		1.50	+3750
Vitamin K (mcg)	0.20		400.00	+200,000

## 32. Restoring Microbiota—Key to Success?

Studies of the critically ill, especially those with systemic inflammatory reaction syndrome (SIRS), report severe dysbiosis. When compared with healthy volunteers, they often have 10,000 times fewer total anaerobes, including the “beneficial” Bifidobacterium and Lactobacillus, and 100 times more “pathogenic” bacteria, such as Staphylococcus bacteria. The content in the gut of organic acids, in general, but butyric and propionic acids, in particular, are severely reduced. Recent studies report the production of “mucosa-tightening” butyric acid as almost extinct (from 16.6 ± 6.7 to 0.9 ± 2.3) [72,73,74].

The incidence of organ failure and ICU mortality is reported to be significantly higher in patients with profound reductions in size and diversity of microbiota, especially when associated with a massive presence of enterococci and with the use of antibiotics, especially clindamycin [186]. Information like this provides strong support to efforts to prevent dysfunctioning microbiota, “dysbiosis” and, when needed—as it always is for critically ill patients—strong and forceful efforts to restore homeostasis in microbiota, the ideal state of balance being called “eubiosis”. Recent cutting-edge results from the supplementation of synbiotics to postoperative and critically ill patients as discussed earlier, support recommendations to routinely supplement specific LAB and fibers (synbiotics) to a wide group of patients undergoing major medical or surgical treatments or suffering from polytrauma or medical emergencies such as acute pancreatitis or myocardial infarction. For patients who cannot tolerate enteral feeding, administration of synbiotics by enemas with live LAB may be a treatment option. It is important to remember that most patients do not die of their disease but from a disordered physiology caused by either the disease or, as often happens, by the treatments. 
